# Effect of Pre- and In-Hospital Delay on Reperfusion in Acute Ischemic Stroke Mechanical Thrombectomy

**DOI:** 10.1161/STROKEAHA.120.030208

**Published:** 2020-09-16

**Authors:** Johannes Kaesmacher, Basel Maamari, Thomas R. Meinel, Eike I. Piechowiak, Pascal J. Mosimann, Pasquale Mordasini, Martina Goeldlin, Marcel Arnold, Tomas Dobrocky, Tobias Boeckh-Behrens, Maria Berndt, Patrik Michel, Manuel Requena, Amel Benali, Laurent Pierot, Vitor Mendes Pereira, Grégoire Boulouis, Alex Brehm, Peter B. Sporns, Johanna M. Ospel, Jan Gralla, Urs Fischer

**Affiliations:** 1 University Institute of Diagnostic and Interventional Neuroradiology (J.K., E.I.P., P.J.M., P. Mordasini, T.D., J.G.), University Hospital Bern, Inselspital, University of Bern, Switzerland.; 2 University Institute of Diagnostic and Interventional and Pediatric Radiology (J.K.), University Hospital Bern, Inselspital, University of Bern, Switzerland.; 3 Department of Neurology (B.M., T.R.M., M.G., M.A., U.F.), University Hospital Bern, Inselspital, University of Bern, Switzerland.; 4 Department of Diagnostic and Interventional Neuroradiology, Klinikum rechts der Isar, Technical University Munich, Germany (T.B.-B., M.B.).; 5 Department of Neurology, CHUV Lausanne, Switzerland (P. Michel).; 6 Department of Neurology, Vall d’Hebron University Hospital, Barcelona, Spain (M.R.).; 7 Department of Neuroradiology, CHU Montpellier, France (A. Benali).; 8 Department of Neuroradiology, CHU Reims, France (L.P.).; 9 Joint Department of Medical Imaging and Division of Neurosurgery, Toronto Western Hospital, University of Toronto, ON, Canada (V.M.P.).; 10 Department of Neuroradiology, Université Paris Descartes, Sainte Anne Hospital, France (G.B.).; 11 Department of Neuroradiology (A. Brehm, P.B.S.), University Hospital Basel, Switzerland.; 12 Department of Radiology (J.M.O.), University Hospital Basel, Switzerland.; 13 Department of Clinical Neuroscience, University of Calgary, Canada (J.M.O.).

**Keywords:** odds ratio, reperfusion, thrombectomy, workflow

## Abstract

**Background and Purpose:**

Post hoc analyses of randomized controlled clinical trials evaluating mechanical thrombectomy have suggested that admission-to-groin-puncture (ATG) delays are associated with reduced reperfusion rates. Purpose of this analysis was to validate this association in a real-world cohort and to find associated factors and confounders for prolonged ATG intervals.

**Methods:**

Patients included into the BEYOND-SWIFT cohort (Bernese-European Registry for Ischemic Stroke Patients Treated Outside Current Guidelines With Neurothrombectomy Devices Using the Solitaire FR With the Intention for Thrombectomy; https://www.clinicaltrials.gov; Unique identifier: NCT03496064) were analyzed (n=2386). Association between baseline characteristics and ATG was evaluated using mixed linear regression analysis. The effect of increasing symptom-onset-to-admission and ATG intervals on successful reperfusion (defined as Thrombolysis in Cerebral Infarction [TICI] 2b-3) was evaluated using logistic regression analysis adjusting for potential confounders.

**Results:**

Median ATG was 73 minutes. Prolonged ATG intervals were associated with the use of magnetic resonance imaging (+19.1 [95% CI, +9.1 to +29.1] minutes), general anesthesia (+12.1 [95% CI, +3.7 to +20.4] minutes), and borderline indication criteria, such as lower National Institutes of Health Stroke Scale, late presentations, or not meeting top-tier early time window eligibility criteria (+13.8 [95% CI, +6.1 to +21.6] minutes). There was a 13% relative odds reduction for TICI 2b-3 (adjusted odds ratio [aOR], 0.87 [95% CI, 0.79–0.96]) and TICI 2c/3 (aOR, 0.87 [95% CI, 0.79–0.95]) per hour ATG delay, while the reduction of TICI 2b-3 per hour increase symptom-onset-to-admission was minor (aOR, 0.97 [95% CI, 0.94–0.99]) and inconsistent regarding TICI 2c/3 (aOR, 0.99 [95% CI, 0.97–1.02]). After adjusting for identified factors associated with prolonged ATG intervals, the association of ATG delay and lower rates of TICI 2b-3 remained tangible (aOR, 0.87 [95% CI, 0.76–0.99]).

**Conclusions:**

There is a great potential to reduce ATG, and potential targets for improvement can be deduced from observational data. The association between in-hospital delay and reduced reperfusion rates is evident in real-world clinical data, underscoring the need to optimize in-hospital workflows. Given the only minor association between symptom-onset-to-admission intervals and reperfusion rates, the causal relationship of this association warrants further research.

**Registration:**

URL: https://www.clinicaltrials.gov. Unique identifier: NCT03496064.

## Abstract 2

Supplemental Digital Content is available in the text.

Achieving successful and ideally complete reperfusion is the most important modifiable predictor of good outcome in patients with acute ischemic stroke undergoing mechanical thrombectomy.^1–4^ A recent analysis of the HERMES (Highly Effective Reperfusion Evaluated in Multiple Endovascular Stroke Trials) collaboration suggested that the chances of achieving reperfusion decrease with prolonged admission-to-groin (ATG) intervals, with an estimated relative decrease in the odds of successful reperfusion of 22% per hour of in-hospital delay.^5^

No significant effect on successful reperfusion was found for symptom-onset-to-groin-puncture (STG) intervals, however.^5^ This discrepancy was attributed mostly to the strict inclusion criteria of the randomized controlled trials and uncertainties regarding the precision of the symptom onset, hence diluting a possibly significant effect of time elapsed from symptom onset.^5^

In the current study, we aimed to quantify the effect of symptom-to-admission (STA), ATG, and STG intervals on rates of successful reperfusion in a real-world registry with less restrictive inclusion criteria and performed explorative analysis regarding potential confounders.

## Methods

The data that support the findings of this study are available from the corresponding author upon reasonable request and after clearance by the local ethics committee.

### BEYOND-SWIFT Registry

The BEYOND-SWIFT (Bernese-European Registry for Ischemic Stroke Patients Treated Outside Current Guidelines With Neurothrombectomy Devices Using the Solitaire FR With the Intention for Thrombectomy) is an investigator-initiated, international, multicenter observational registry evaluating the outcome of mechanical thrombectomy patients (https://www.clinicaltrials.gov; Unique identifier: NCT03496064). Details of the registry have been published previously.^6^ In short, 7 comprehensive stroke centers included patients treated with a Medtronic market-released neurothrombectomy device (applied as first-line device) for patients presenting with large vessel occlusion acute ischemic stroke. Table I in the Data Supplement provides an overview of included patients and rates of available follow-up data for each center. Written informed consent was obtained from patients, or the institutional review board waived the need for patient consent, according to regulations at each center/country (Table I in the Data Supplement). In Bern, ethical approval was given for pooling and analysis of the registry data (Kantonale Ethikkommission Bern, Bern, Switzerland; Local Ethics Committee Study Identifier: 2018-00766). For the present analysis, we also included 437 patients from another German center.^7^

Of 2397 patients included into the registry, 2127 were treated for anterior circulation large vessel occlusion strokes. Of these, there were 1953 patients with full documentation of STA, ATG, and STG, and 1949 had available angiography runs to evaluate reperfusion success. Using the same selection criteria for the additional German center, of 547 patients, 437 were included.

### Variables and Outcomes

Site of occlusion was classified into intracranial internal carotid artery, carotid T/L, and first/second segment of the middle cerebral artery. Reperfusion success was evaluated applying the extended Thrombolysis in Cerebral Infarction (TICI) scale with TICI 2b defined as ≥50% reperfusion of the initially hypoperfused target territory and TICI 2c defined as “near-complete perfusion except for slow flow in a few distal cortical vessels or presence of small distal cortical emboli.”^8,9^ Reperfusion success was rated by an independent research fellow or was operator adjudicated depending on the centers’ standards. Alberta Stroke Program Early CT Scores (ASPECTS) were evaluated on noncontrast admission computed tomography images or diffusion-weighted imaging–based ASPECTS when patients underwent magnetic resonance imaging (MRI). Slow and fast progressors were dichotomized according to a median split of ASPECTS decay (estimated as ASPECTS regions infarcted/time from STA). Interventional characteristics were recorded including interventional technique, maneuver count, interventional complications, and time from groin puncture to reperfusion. Functional outcome was assessed at 3 months after the index event using the modified Rankin Scale (mRS), with mRS ≤2 defined as good functional outcome. Symptomatic intracranial hemorrhage was defined according to ECASS-II (European Co-Operative Acute Stroke Study-II) definition.^10^ Primary outcome of this analysis was the rate of TICI 2b-3 with strata of ATG intervals. Secondary outcomes were the evaluation of a potential association between STA and STG and rates of TICI 2b-3. Explorative analyses were performed regarding rates of TICI 2c/3, rates of first-pass TICI 2c/3, periprocedural complications, utilizing >3 maneuvers, and occurrence of symptomatic intracranial hemorrhage with strata of ATG intervals.

### Descriptive Statistics

Continuous variables are presented as mean±SD or median and interquartile range (IQR). Frequency counts are shown as percentage and n/N.

### Logistic Regression Analysis

The effect of STA, ATG, and STG on TICI 2b-3 was evaluated using multivariate binary logistic regression analysis with STA/ATG included as continuous predictor variable and TICI 2b-3 as outcome variable. Mixed logistic regression analyses with random effects were omitted because preanalysis did not provide evidence for model superiority against simple logistic regression analysis (Table II in the Data Supplement). In the first logistic regression model (model A), analyses were adjusted for age, sex, occlusion location (categorical variable: intracranial internal carotid artery/carotid T/L as reference versus M1 versus M2), and treatment with intravenous tPA (tissue-type plasminogen activator), as described previously.^5^ Subgroup analyses were conducted for slow versus fast progressor in STA analyses and for computed tomography versus MRI in ATG analyses, utilizing interaction terms. Modeled predicted probabilities of TICI 2b-3 with increasing STA or ATG were displayed treating all other variables in the model at mean (continuous variables) or at balance (categorical variables). In model B, we incorporated all variables associated with ATG from the analysis outlined below together with stroke etiologic cause (according to the Trial of ORG 10172 in Acute Stroke Treatment criteria), interventional technique,^11^ and year of patient treatment (see Table III in the Data Supplement for a model overview). We also ran an additional sensitivity analysis including periprocedural complications and number of maneuvers into the model (model B*). All logistic regression models are adjusted for center. Outputs of logistic regression analyses are generally displayed as adjusted odds ratio (aOR) per 60-minute increase and corresponding 95% CIs.

### Mixed Linear Regression Analysis

To find associations between baseline characteristics and prolonged ATG intervals, a mixed linear regression analysis was performed because a log-likelihood test revealed improved model fit as opposed to a simple linear regression model. Age, sex, direct admission versus transfer, functional dependence (mRS score >2) before the index event, admission National Institutes of Heath Stroke Scale (NIHSS) scores, STA, imaging modality, ASPECTS, intracranial occlusion, tandem occlusion, general anesthesia, intravenous tPA treatment, and year of treatment were included in the model. Alternatively, we implemented treatment eligibility according to early time window American Heart Association (AHA)/American Stroke Association (ASA) criteria, omitting variables included into eligibility criteria (age, admission NIHSS, etc). Early time window eligibility according to AHA/ASA criteria was defined as a compound criterion of meeting prestroke mRS score <2, internal carotid artery or M1 occlusion, age >18 years, NIHSS ≥6, ASPECTS ≥6, and time to groin ≤6 hours.^12^ Center site was implemented as a random-effects variable.

Significance level was set to α=0.05. All tests are 2 sided. All analyses were conducted using STATA (v 15.1; Stata Corp, TX).

## Results

### Cohort

Two thousand three hundred eighty-six patients were included (median age, 74.7 years; IQR, 62.2–82.0; 51.2% women; Table 1), of which 2008 (84.2%) were successfully reperfused (TICI 2b-3, including 54.4% with TICI 2c/3 reperfusions). Rates of successful reperfusion and TICI 2c/3 differed across centers (Figure I in the Data Supplement) and by year of patient treatment (odds ratio per year increase after 2015, 1.16 [95% CI, 1.05–1.28]; Figure II in the Data Supplement). Additionally, we observed an association between interventional technique applied and reperfusion success, with the highest rates of successful reperfusion or TICI 2c/3 observed in patients treated with stent retriever and a balloon guide catheter (Figure III in the Data Supplement). Median STA delay was 150 minutes (IQR, 72–265 minutes), whereas median ATG interval was 73 minutes (IQR, 47–102 minutes). The relative percentage of ATG from STG was 33.0% (IQR, 16.4%–52.6%). Occlusion site was mostly M1 (57.2%, 1365 of 2386), followed by intracranial internal carotid artery/carotid T/L (26.2%, 625 of 2386) and M2 occlusions (16.6%, 396 of 2386).

**Table 1.**
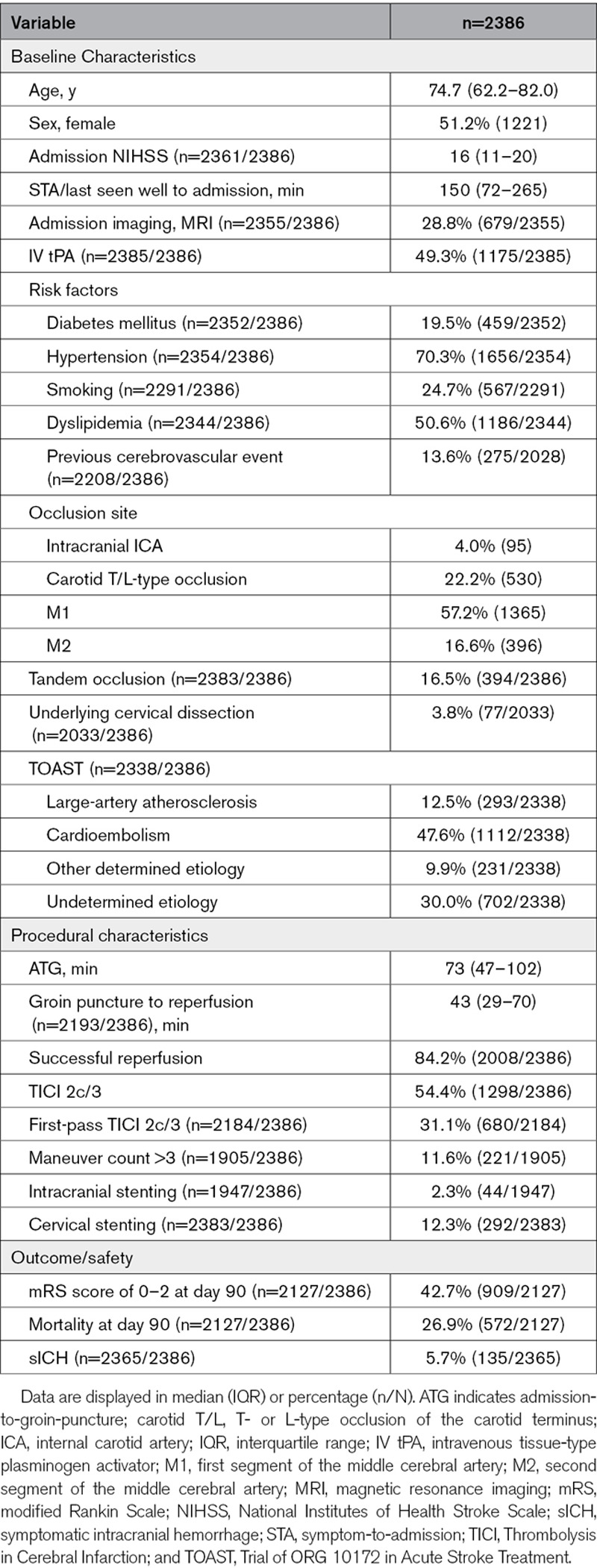
Study Cohort

### Effect of STA and ATG on Successful Reperfusion

**Figure 1.**
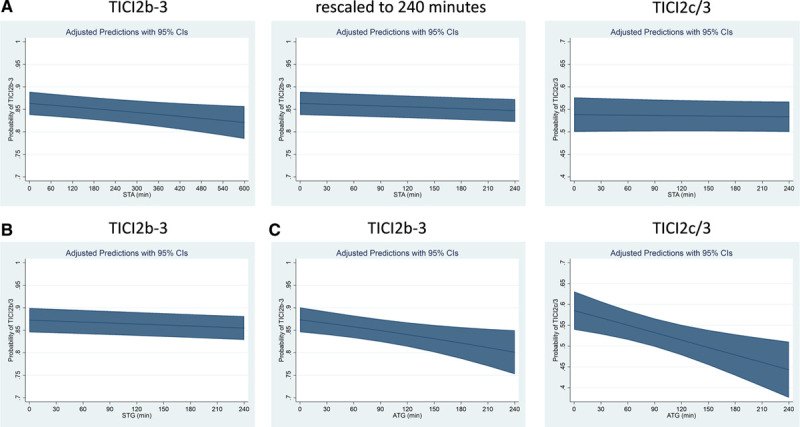
**Association of symptom-onset-to-admission (STA) and admission-to-groin-puncture (ATG) intervals with the probability of achieving Thrombolysis in Cerebral Infarction (TICI) 2b-3 or 2c/3.** Adjusted predicted probabilities of TICI 2b-3 or 2c/3 according to STA, symptom-onset-to-groin puncture (STG), and ATG intervals in minutes (see Methods). **A**, A small association of increasing STA with decreasing odds of achieving TICI 2b-3 was found (adjusted odds ratio [aOR], 0.96 [95%, 0.94–0.99] per hour) while no statistically significant association between STA and the odds of achieving TICI 2c/3 was observed (aOR, 0.99 [95% CI, 0.97–1.02] per hour). **B**, A small association of increasing STG with decreasing odds of achieving TICI 2b-3 was found (aOR, 0.96 [95%, 0.94–0.99] per hour). **C**, With increasing ATG, there was a strong reduction in the rates of TICI 2b-3 (aOR, 0.87 [95% CI, 0.79–0.96] per hour), corresponding to a 13% reduction in the odds of TICI 2b-3 per in-hospital hour delay. This association was also stable when considering TICI 2c/3 as relevant end point (aOR, 0.87 [95% CI, 0.79–0.95] per hour).

Overall, there was a small reduction in the likelihood of achieving TICI 2b-3 with increasing STA (aOR, 0.97 [95% CI, 0.94–0.99] per hour; Table 2; Figure 1A). This association appeared stronger in fast progressors (aOR, 0.86 [95% CI, 0.79–0.95] per hour), without reaching statistical significance on interaction analysis (*P* for interaction, 0.081). With increasing ATG, there was a strong reduction in the rates of TICI 2b-3 (aOR, 0.87 [95% CI, 0.79–0.96] per hour; Figure 1C), corresponding to a 13% reduction in the odds of TICI 2b-3 per in-hospital hour delay. This association appeared more pronounced in patients undergoing MRI (aOR, 0.77 [95% CI, 0.65–0.91]), without reaching significance on interaction analysis (*P* for interaction, 0.102).

**Table 2.**
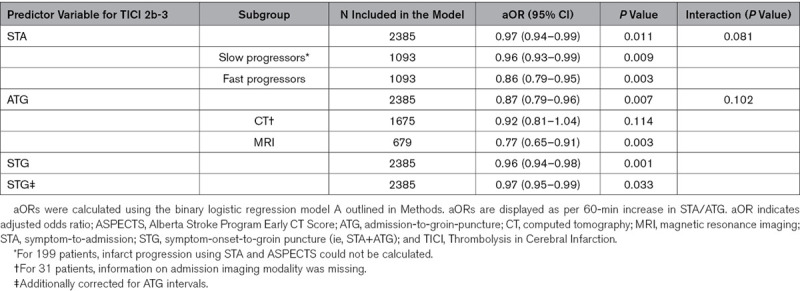
Logistic Regression Analysis With STA/ATG as Predictor Variable and TICI 2b-3 as Outcome Variable

For ATG, these association remained unchanged when considering TICI 2c/3 as relevant interventional end point (aOR, 0.87 [95% CI, 0.79–0.95]; Figure 1C), while no significant association of STA with rates of TICI 2c/3 was found (aOR, 0.99 [95% CI, 0.97–1.02]; Figure 1A).

For STG intervals, a significant association with TICI 2b-3 was found (Figure 1B), but this was partially attributed to the association of ATG intervals with TICI 2b-3 (see STG* in Table 2).

### Association of ATG With Secondary Interventional Outcomes

**Figure 2.**
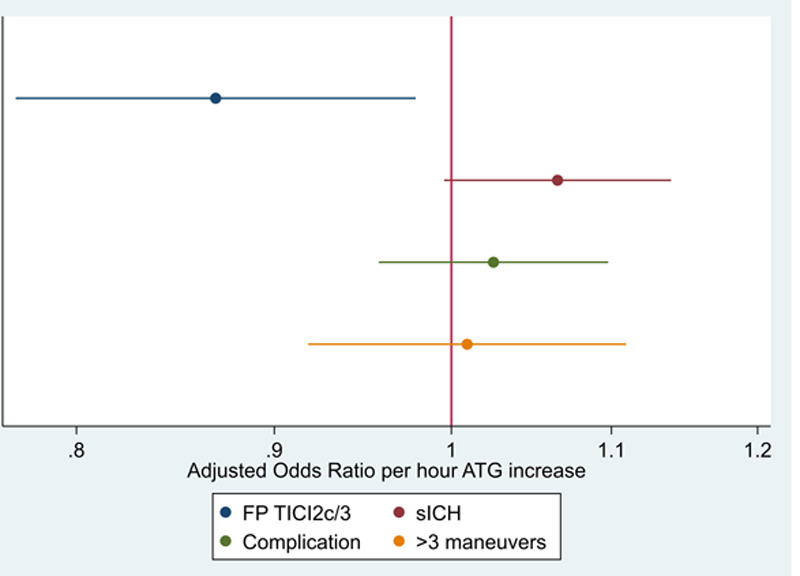
**Association of admission-to-groin-puncture (ATG) intervals with secondary procedural outcomes.** A significant effect of ATG was found regarding rates of first-pass Thrombolysis in Cerebral Infarction (FP TICI) 2c/3 (adjusted odds ratio, 0.87 [95% CI, 0.77–0.98]), while no significant associations were observed for all other secondary outcomes. sICH indicates symptomatic intracranial hemorrhage.

Every hour decrease in ATG was associated with reduced rates of first-pass TICI 2c/3 (aOR, 0.87 [95% CI, 0.77–0.98]). There was no statistically significant association of ATG with other interventional characteristics, including complications, rates of symptomatic intracranial hemorrhage, and utilization of >3 maneuvers (Figure 2).

### Factors Associated With ATG

**Figure 3.**
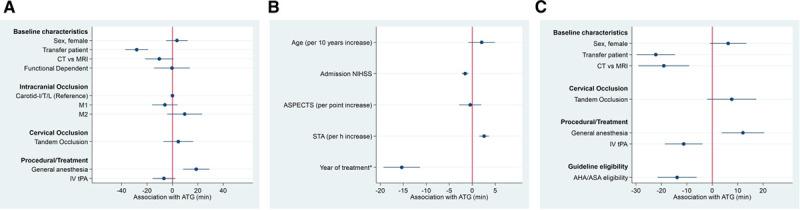
**Adjusted differences in admission-to-groin-puncture (ATG) intervals according to baseline and procedural variables.** **A**, Categorical variables (effect scale, −60 to 60 min), patients receiving magnetic resonance imaging (MRI) or general anesthesia had increased ATG. **B**, Continuous variables (effect scale, −10 to 10 min), patients presenting late, patients with lower National Institutes of Health Stroke Scale (NIHSS), and patients treated in earlier years had increased ATG. **C**, Same model but functional dependence, age, Alberta Stroke Program Early CT Score (ASPECTS), admission NIHSS, and symptom-to-admission (STA) replaced by a compound variable of meeting American Heart Association (AHA)/American Stroke Association (ASA) guideline indication criteria. Patients meeting AHA/ASA guideline indication criteria on average had 14 min shorter ATG. CT indicates computed tomography; IV tPA, intravenous tissue-type plasminogen activator; M1, first segment of the middle cerebral artery; and M2, second segment of the middle cerebral artery. *Year of treatment implemented as continuous variable as per year increase since 2015.

Of 2386 patients, 2240 were included in the random-effects linear regression analysis for identifying factors associated with ATG (Figure 3A and 3B). Transfer admissions (−28.1 [95% CI, −37.2 to −19.1] minutes), computed tomography versus MRI (−10.3 [95% CI, −21.4 to +0.8] minutes in the model without the compound AHA/ASA eligibility variable and −19.1 [95% CI, −29.1 to −9.1] minutes in the model with the compound AHA/ASA eligibility variable), higher admission NIHSS (−1.5 minutes per point increase [95% CI, −2.3 to −0.8 minutes]), and patient treatment in recent years (−15.3 minutes per year increase since 2015 [95% CI, −19.3 to −11.4 minutes]) were associated with shorter ATG intervals. In contrary, late presentation (+2.6 minutes per minute presentation delay [95% CI, +1.5 to +3.6 minutes]) and use of general anesthesia (+18.7 [95% CI, 8.4–29.0] minutes) were associated with longer ATGs. Implementing the AHA/ASA eligibility criteria to the model, conformance with the AHA/ASA early time window eligibility criteria was associated with shorter ATG intervals (−13.8 [95% CI, −21.6 to −6.1] minutes; Figure 3C).

### Sensitivity Analyses Utilizing Refined Models for the Effect of ATG

**Figure 4.**
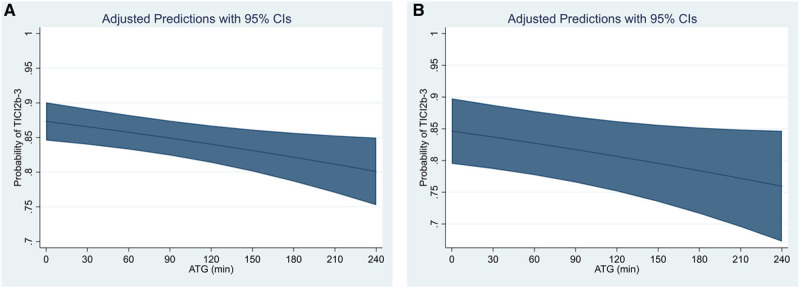
**Association of admission-to-groin-puncture (ATG) intervals with probability of Thrombolysis in Cerebral Infarction (TICI) 2b-3 in various models.** **A**, Adjusted predicted probabilities of TICI 2b-3 with respect to increasing ATG intervals using model A (corresponding to the model used by Bourcier et al). **B**, Adjusted predicted probabilities of TICI 2b-3 applying a refined logistic regression model B, additionally adjusting for factors associated with increased ATG, stroke etiologic cause, interventional technique, and year of treatment (model B).

We included 1822 of 2386 patients in the refined model B. When incorporating factors associated with prolonged ATG intervals, together with adjustment for stroke etiology, year of patient inclusion, and interventional technique, the effect of ATG on rates of TICI 2b-3 could still be detected, yielding a 13% relative decrease in the odds of TICI 2b-3 per hour of ATG delay (aOR, 0.87 [95% CI, 0.76–0.99]; Figure 4). Additionally implementing the number of maneuvers and periprocedural complications as covariates did not significantly change this association (model B*; aOR, 0.74 [95% CI, 0.59–0.92]).

### Clinical Outcomes With Respect to Admission to Reperfusion Intervals

There was a 19% relative odds reduction in achieving mRS score of 0 to 2 for every hour in-hospital delay (aOR, 0.81 [95% CI, 0.73–0.90]). There was no interaction regarding this effect and occlusion site (*P*=0.64) or AHA/ASA guideline eligibility (*P*=0.51). This effect was less pronounced when considering the time elapsed from symptom onset (aOR per hour delay, 0.96 [95% CI, 0.93–0.99]).

## Discussion

This registry analysis has the following main findings: (1) the association of a reduced rate of TICI 2b-3 with increasing ATG is evident in real-world clinical data with a comparable effect size to what has been reported from randomized controlled trial data; (2) in contrast, the association between STA and TICI 2b-3 appears considerably weaker and is not consistent for other interventional end points (eg, TICI 2c/3); (3) patients with borderline indications not meeting early time window AHA/ASA guideline criteria were more likely to have prolonged ATG intervals, probably reflecting difficulties in treatment decision-making, which may also relate to pursuing the goal of TICI 2b-3 less rigorously; (4) even after correcting for such factors and other confounders (interventional technique, year of patient treatment) associated with prolonged ATG intervals, the effect of ATG on TICI 2b-3 remained statistically robust; (5) in high-volume centers ATG intervals are quiet long, and associated factors like the use of MRI or general anesthesia, as well as patient characteristics associated with delays, have been identified as potential targets for improvement programs.

Achieving successful reperfusion remains the most important modifiable predictor of outcome in patients presenting with acute ischemic stroke due to large vessel occlusion. Accordingly, identifying factors associated with decreased rates of successful reperfusion is important. In our study, each hour of ATG delay was associated with a relative decrease of 13% in the odds of TICI 2b-3, comparing slightly lower than the 22% odds reduction published by the HERMES collaboration.^5^ Our findings support that this association is not only true for selected randomized controlled trial patients but also tangible in a real-world cohort of patients with less restrictive inclusion criteria. We found a weak association between STA and rates of TICI 2b-3 and observed a significant impact of STG interval on TICI 2b-3, although this effect seems mainly mediated by the association of ATG with TICI 2b-3.

One of the most important findings of the multicenter registry is that ATG intervals are quite long, with a median delay of 73 minutes. Allowing for varying effect sizes across centers, we were able to identify relevant factors associated with prolonged ATG intervals. These included the use of MRI and general anesthesia, as well as patient admission characteristics, such as low admission NIHSS, late presentation, and not meeting early time window AHA/ASA guideline criteria. Contrary to previous findings,^13^ administration of intravenous tPA did not prolong the ATG interval. Even though treatment decisions in patients not meeting guideline criteria should be individualized, there seems to be a significant delay in ATG intervals for those patients. To reduce such delays is not only important for improving patients outcome but should be kept in mind when evaluating the outcome of patients presenting with borderline criteria subjected to mechanical thrombectomy. This real-world data indicate that in these patients, potential beneficial effects may be masked by associated delays in ATG intervals and respective lower rates of achieving TICI 2b-3. Corroborating previous studies, the use of admission MRI was associated with in-hospital delays,^14,15^ further advocating the need for quality improvement program regarding sequence efficiency, as described recently.^16^

Several possible causal relations between prolonged ATG intervals and reduced rates of TICI 2b-3 have previously been discussed,^5^ such as thrombus modification over time (volume/extension,^17^ histological features^18^), which might influence mechanical reperfusion efficacy.^19,20^ At first sight, however, this may be contradictive to the only small association between STA and TICI 2b-3 because STA does usually constitute by far more of the time interval elapsed between symptom onset and the start of the intervention (≈2/3 in our cohort). Bourcier et al argued that the effect of STA may be diluted by uncertainties regarding the exact time point of symptom onset. Additionally, the authors argued that the effect of STA may be confounded by strict inclusion criteria of the randomized controlled trials (ie, only including patients with good collaterals, small infarct cores, incomplete occlusion pattern).^5^ The latter phenomenon concerning the effect of time on functional outcome, often referred to as the time-reset effect, is indeed well known: admission/imaging-to-groin intervals have a stronger association with outcome, as opposed to STA intervals, as also observed in this registry.^21^ Given the less restrictive selection criteria of our patient cohort, however, one would expect this bias to be weaker, thereby potentially unmasking an underlying association.

Our results provide evidence that prolonged ATG intervals are associated with borderline selection criteria. In these cases, it is reasonable to assume that decision-making is generally prolonged. The grit to achieve successful reperfusion in borderline indications may perhaps also diminish, which might explain decreased TICI 2b-3 with increasing ATG. It is noteworthy that the association of ATG with TICI 2b-3 persisted after adjusting for confounding factors associated with prolonged ATG intervals. However, this does not necessarily imply the absence of more ill-defined residual confounding factors related to more complex decision-making, such as difficult vascular anatomy, questionable life expectancy/comorbidities (cancer and dementia), or the search for a second/third opinion that may impede the dedication of subsequently pursuing TICI 2b-3 as rigorously as in more clear-cut cases.

Last, there is a paralleling development regarding improvements in rates of TICI 2b-3 and shorter ATG intervals in recent years following publication of the large randomized controlled trials in 2015. Hence, an association of ATG with TICI 2b-3 may simply reflect this technical development or alternatively is a proxy reflecting that more experienced centers with presumingly shorter ATG also have higher rates of TICI 2b-3. However, correcting for center and years of patient inclusion yielded a stable point estimate and 95% CI, suggesting that the association of ATG with rates of TICI 2b-3 is not merely explained by such technical developments or center-specific considerations.

Until further evidence becomes available, the causal relationship between prolonged ATG intervals and reduced rates of TICI 2b-3 with only a minor impact of STA remains elusive. More focus is needed on decision-making and workflow factors related to patients’ characteristics and prolonged ATG intervals. Before such evidence becomes available, we do not know to what extent dawdling diminishes reperfusion or if prolonged ATG intervals relate to patient characteristics, where reperfusion is less often achieved or less rigorously pursued to be achieved.

### Strengths and Limitations

Strengths of this analysis are the large cohort of patients and increased sample sizes in subgroups thereby increasing statistical power, as well as adequate modeling, enabling to explore factors associated with prolonged ATG intervals while correcting for confounders. Limitations of this study include a high percentage of incomplete complete workflow metrics and the retrospective nature of the registry. Most importantly, reperfusion success was not core laboratory adjudicated, impeding generalizability of the findings and comparability across centers. While this may be especially relevant for differentiating TICI 2b and TICI 3,^22^ the agreement between operator and core laboratory for dichotomized TICI scores (ie, TICI 0/1/2a versus TICI 2b-3) is usually substantial^22^—an argument that supports the validity of our findings. Moreover, the association between prolonged ATG and reduced rates of, for example, TICI 2c/3 or first-pass TICI 2c/3 are interesting but should be handled with caution, since these variables were not defined as the primary outcome and, as secondary explorative analyses, are susceptible to α-error inflation.

### Conclusions

There is a great potential to reduce ATG, and potential targets for improvement can be deduced from observational data. The association between in-hospital delay and reduced reperfusion rates is evident in real-world clinical data, underscoring the need to optimize in-hospital workflows. Given the only minor association between STA and reperfusion rates and controversial pathophysiological considerations, the causal relationship of this association warrants further research.

## Sources of Funding

This study was supported by Medtronic (Dublin, Ireland). Medtronic did not take part in the conception, design, or article draft of this study. The work of Dr Kaesmacher was supported by the Swiss Academy of Medical Sciences/Bangerter Foundation and the Swiss Stroke Society.

## Disclosures

Dr Fischer reports grants from Medtronic during the conduct of the study, grants from Medtronic, and other from Medtronic, Stryker, and CSL Behring outside the submitted work. Dr Gralla is a global principal investigator of STAR (Solitaire FR Thrombectomy for Acute Revascularisation), Clinical Event Committee member of the PROMISE study (Prospective, Multicenter, Observational, Single-Arm European Registry on the ACE Reperfusion Catheters and the Penumbra System in the Treatment of Acute Ischemic Stroke; Penumbra), and a principal investigator and consultant for the SWIFT DIRECT study (Solitaire With the Intention for Thrombectomy Plus Intravenous tPA Versus DIRECT Solitaire Stent-Retriever Thrombectomy in Acute Anterior Circulation Stroke; Medtronic) and receives Swiss National Science Foundation grants for magnetic resonance imaging in stroke. Dr Pierot reports personal fees from Balt, Phenox, and Microvention outside the submitted work. Dr Kaesmacher reports grants from Swiss Academy of Medical Sciences/Bangerter Foundation, Swiss Stroke Society, and Clinical Trial Unit Bern during the conduct of the study. Dr Michel reports grants from the Swiss National Science Foundation and the Swiss Heart Foundation during the conduct of the study; grants from European Thrombosis Investigator-Initiated Research program (Bristol-Myers Squibb/Pfizer), and personal fees from Medtronic used for research, outside the submitted work. Dr Arnold reports personal fees from Bayer, Bristol-Myers Squibb, Medtronic, Amgen, Daiichi Sankyo, Nestlé Health Sciences, Boehringer Ingelheim, and Covidien during the conduct of the study. Dr Mendes Pereira reports personal fees from Medtronic and Stryker during the conduct of the study. The other authors report no conflicts.

## Supplemental Materials

Tables I–III

Figures I–III

Reference 6

## Supplementary Material


