# Supplementary material for: Effect of Pre- and In-Hospital Delay on Reperfusion in Acute Ischemic Stroke Mechanical Thrombectomy
Source: Stroke. 2020 Sep 16;51(10):2934–42. doi: 10.1161/STROKEAHA.120.030208 (PMC7523579; doi:10.1161/STROKEAHA.120.030208)
Supplement: Supplementary file 1 [file str-51-2934-s001.pdf]

Effect of pre- and in-hospital delay on reperfusion in acute ischemic stroke mechanical thrombectomy

Running title: Does dawdling diminish reperfusion?

Johannes Kaesmacher MD[a,b*], Basel Maamari MD[c*], Thomas R. Meinel MD[c], Eike I Piechowiak MD[a], Pascal J Mosimann MD[a], Pasquale Mordasini MD[a], Martina Goeldlin MD[c], Marcel Arnold MD[c], Tomas Dobrocky MD[a], Tobias Boeckh-Behrens MD[d], Maria Berndt MD[d], Patrik Michel MD[e], Manuel Requena MD[f], Amel Benali MSc[g], Laurent Pierot MD PhD[h], Vitor M Pereira MD PhD[i], Grégoire Boulouis MD MSc[j], Alex Brehm MD[k], Peter B Sporns MD[k], Johanna M Ospel MD[l,m], Jan Gralla MD MSc[a**] and Urs Fischer MD MSc[c**] on behalf of the BEYOND-SWIFT Investigators

a University Institute of Diagnostic and Interventional Neuroradiology, University Hospital Bern, Inselspital, University of Bern, Bern, Switzerland
b University Institute of Diagnostic and Interventional and Pediatric Radiology, University Hospital Bern, Inselspital, University of Bern, Bern, Switzerland
c Department of Neurology, University Hospital Bern, Inselspital, University of Bern, Bern, Switzerland
d Department of Diagnostic and Interventional Neuroradiology, Klinikum rechts der Isar, Technical University Munich, Munich, Germany
e Department of Neurology, CHUV Lausanne, Lausanne, Switzerland
f Department of Neurology, Department of Neurology, Vall d'Hebron University Hospital, Barcelona, Spain.
g Department of Neuroradiology, CHU Montpellier, Montpellier, France
h Department of Neuroradiology, CHU Reims, Reims, France
i Joint Department of Medical Imaging and Division of Neurosurgery, Toronto Western Hospital, University of Toronto, Toronto, ON, Canada
j Department of Neuroradiology, Université Paris Descartes, Sainte Anne Hospital, Paris, France
k Department of Neuroradiology, University Hospital Basel, Basel, Switzerland
l Department of Radiology, University Hospital Basel, Basel, Switzerland
m Department of Clinical Neuroscience, University of Calgary, Calgary, Canada

* These authors share first authorship.
** These authors share last authorship.

Corresponding authors:
Urs Fischer, MD, MSc
Department of Neurology
University of Bern
Freiburgstrasse 8
CH-3010, Switzerland
Email: urs.fischer@insel.ch

Figure: 4
Tables: 2
Online Supplement: Yes
Word count abstract: 296

Word count manuscript: 4635

Johannes Kaesmacher, MD
University Institute of Diagnostic and Interventional Neuroradiology
University of Bern
Freiburgstrasse 8
CH-3010, Switzerland
Email: johannes.kaesmacher@insel.ch

Funding: The study was supported by Medtronic (Dublin, Ireland). Medtronic did not take part in the conception, design, or article draft of this study. The work of Dr Kaesmacher was supported by the Swiss Academy of Medical Sciences/Bangerter Foundation and the Swiss Stroke Society.

Full Disclosures (Industry relations) can be found at the end of the manuscript file.

Clinical Trial Registration (observational): ClinicalTrials.gov identifier: NCT03496064

SUPPLEMENTARY MATERIALS

Supplementary Table I – BEYOND-SWIFT overview

**Center**	N	Time period	LVO anterior circulation (ICA, M1, M2)	Lost to follow-up (mRS day 90)	ASPECTS available (LVO anterior circulation)	% MRI as initial imaging modality	ASPECTS grading	Admission and 24h NIHSS	mRS at 90 days	TICI grading	EC approval	Responsible EC
Inselspital Bern, University Hospital Bern, University of Bern, Bern, Switzerland	1317	2010-2018	90.8% (1195/1317)	6.1% (80/1317)	97.7% (1168/1195)	48.5% (637/1317)	Research fellow blinded to clinical data	Board certified stroke neurologists	Stroke neurologists on scheduled clinical visits. Structured telephone interviews if the patient was unable to attend (either by physician or mRS certified stroke nurse).	Operator-measured	Yes	Kantonale Ethik Kommission Bern
Toronto Western Hospital - University Health Network, University of Toronto, Toronto, Canada	60	2014-2017	88.3% (53/60)	0% (0/60)	53/53	1.7% (1/60)	Prospective, by neuroradiologist	Board certified stroke neurologists	Clinical visits at the university hospital. For patients still in rehabilitation facilities, a mRS certified nurse schedules telephone interviews.	Operator-measured	Yes	IRB Toronto
Klinikum rechts der Isar, Technical University Munich, Munich, Germany	206	2009-2017	74.3% (153/206)	18.4% (38/206)	151/153	2.4% (5/206)	Rerospective by neuroradiologist	Board certified stroke neurologists	mRS was evaluated either by face-to-face assessments (by stroke neurologists) or standardized telephone interviews (by certified study nurses).	Operator Measured	Yes	Ethikkommission der medizinischen Fakultät der Technischen Universität München
University Hospital Vall d'Hebron, Barcelona, Spain	418	2010-2017	85.7% (359/419)	20.0% (84/419)	319/360	0% (0/491)	Prospective, by neurologist/neuroradiologist on call	Board certified stroke neurologists	Stroke neurologists on scheduled clinical visits. Structured telephone interviews if unable to attend.	Operator Measured	Yes	CEIC H. Vall d'Hebrond
CHUV, Lausanne University Hospital, Lausanne, Switzerland	139	2012-2017	124/139 (89.2%)	26.6% (37/139)	113/124	0.1% (1/139)	Consensus stroke neurologist and neuroradiologist (not blinded)	Board certified stroke neurologists	mRs was assessed by Rankin-certified physicians at 3 months in the outpatient clinic, or alternatively through a structured telephone interview by Rankin-certified personnel.	Operator-measured	Yes	Commission Ethique de Recherche, Canton de Vaud
Montpellier CHU, University Hospital Montpellier, Montpellier, France	149	2015-2017	97.3% (145/149)	4.0% (6/149)	109/145	82.1% (96/117)	Operator-measured	Board certified stroke neurologists	Stroke neurologists on scheduled clinical visits. Structured telephone interviews if unable to attend.	Operator-measured	(Yes)	Consent was waived owning to the retrospective design (favorable opinion by CNIL)
CHU Reims, University Hospital Reims, Reims, France*	108	2013 - 2017	90.7% (98/108)	0% (0/108)	96/98	92.6% (100/108)	Retrospective, certified neuroradiologist	Board certified stroke neurologists	Stroke physician on clinical visits at university hospital or remote outpatient center.	Retrospective, certified neuroradiologist	(Yes)	Consent was waived owning to the retrospective design
**Non-BEYOND center in this publication**												
University Hospital Göttingen, Göttingen, Germany	547	2015-2019	88.7% (477/547)	15.4% (84/547)	89.1% (425/477)	0% (0/547)	Retrospective certified neuroradiologist	Board certified stroke neurologists	Stroke physician on clinical visits at university hospital or telephone interview	Retrospective, certified neuroradiologist	Yes	Ethik Kommission (UMG)
**Total**	**2944**											

Adapted from Kaesmacheret al with permission. Copyright © 2019, The Authors (CC-BY).[6]

*due to the in- and exclusion criteria of the present study, patient from Reims were not included into the presented analyses (missing work-flow metrics)

Supplementary Table II – Random effect vs simple logistic regression analysis

**Model**	**aOR of ATG on TICI2b/3**	**Log likelihood**	**LR test comparison**
Binary logistic Regression	0.87 (95%-CI 0.79-0.96), P=.002	-1030.104	P>.999
Binary mixed-effects logistic regression	0.89 (95%-CI 0.82-0.98), P=.004	-868.5	

Supplementary Table III – Logistic regression models with TICI2b-3 as dependent variable

Variable	Model A	Model B	Model B*
Age (continuous)	**X**	**X**	**X**
Occlusion site (categorical)	**X**	**X**	**X**
Intravenous thrombolysis (categorical)	**X**	**X**	**X**
Center (categorical)	**X**	**X**	**X**
General anesthesia (categorical)		**X**	**X**
Admission NIHSS (continuous)		**X**	**X**
Symptom-onset to admission (continuous)		**X**	**X**
Transfer vs. direct admission (categorical)		**X**	**X**
AHA/ASA top tier eligibility criteria (categorical)		**X**	**X**
Type of admission imaging (CT vs. MRI, categorical)		**X**	**X**
Interventional technique (categorical)		**X**	**X**
Stroke etiology (categorical)		**X**	**X**
Year of treatment (continuous)		**X**	**X**
Peri-procedural complications (categorical)			**X**
Number of maneuvers (continuous)			**X**

Supplementary Figure I – Reperfusion success stratified by centers

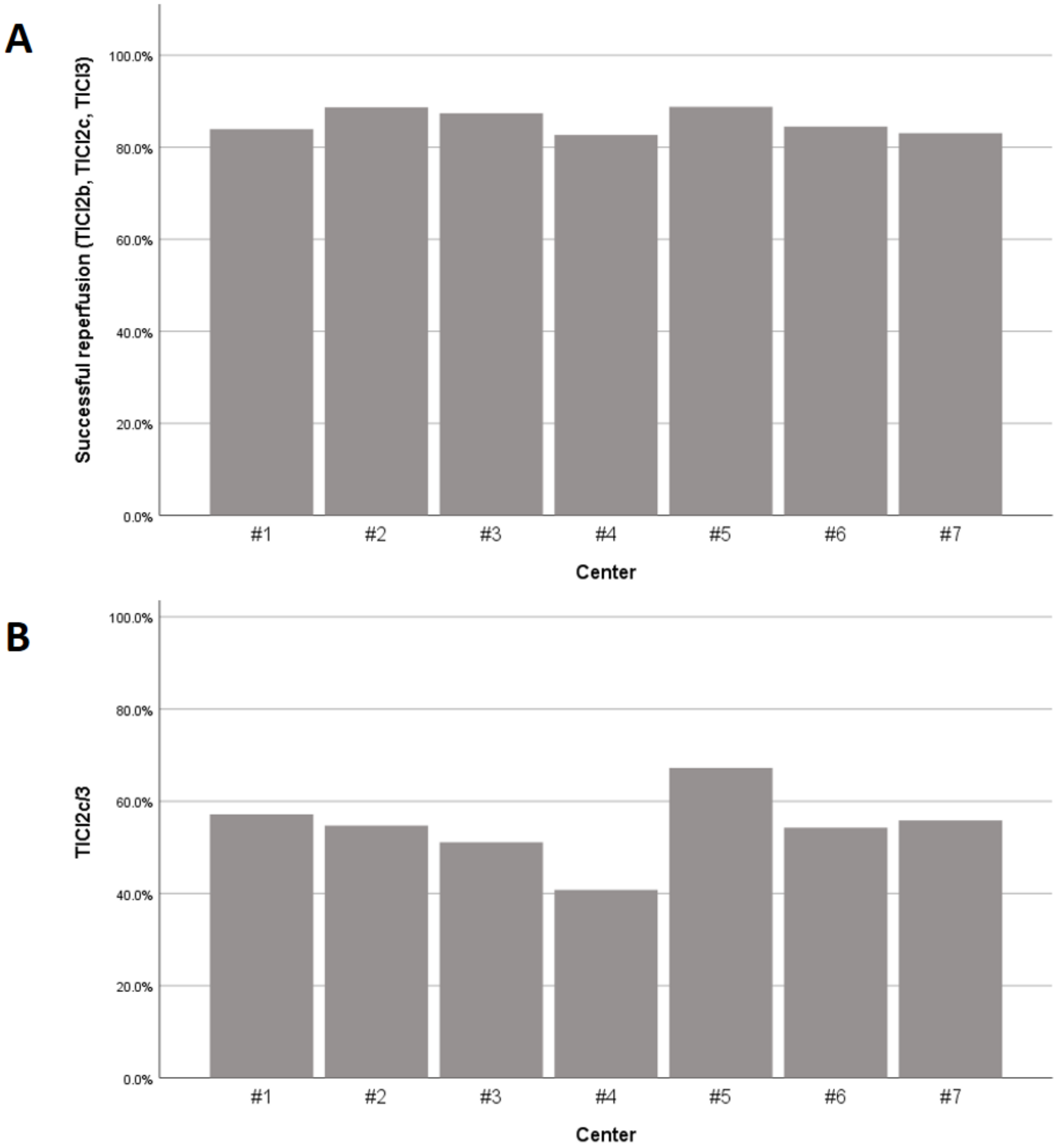

A, Rates of successful reperfusion did not differed significantly across centers (Fisher's exact test, P=0.609, range 82.7-88.8%); **B**, There was a significant difference regarding the rates of TICI2c/3 across centers (P<0.001, range: 32.8%-59.2%). TICI, Thrombolysis in Cerebral Infarction.

Supplementary Figure II – Reperfusion success stratified by year of treatment

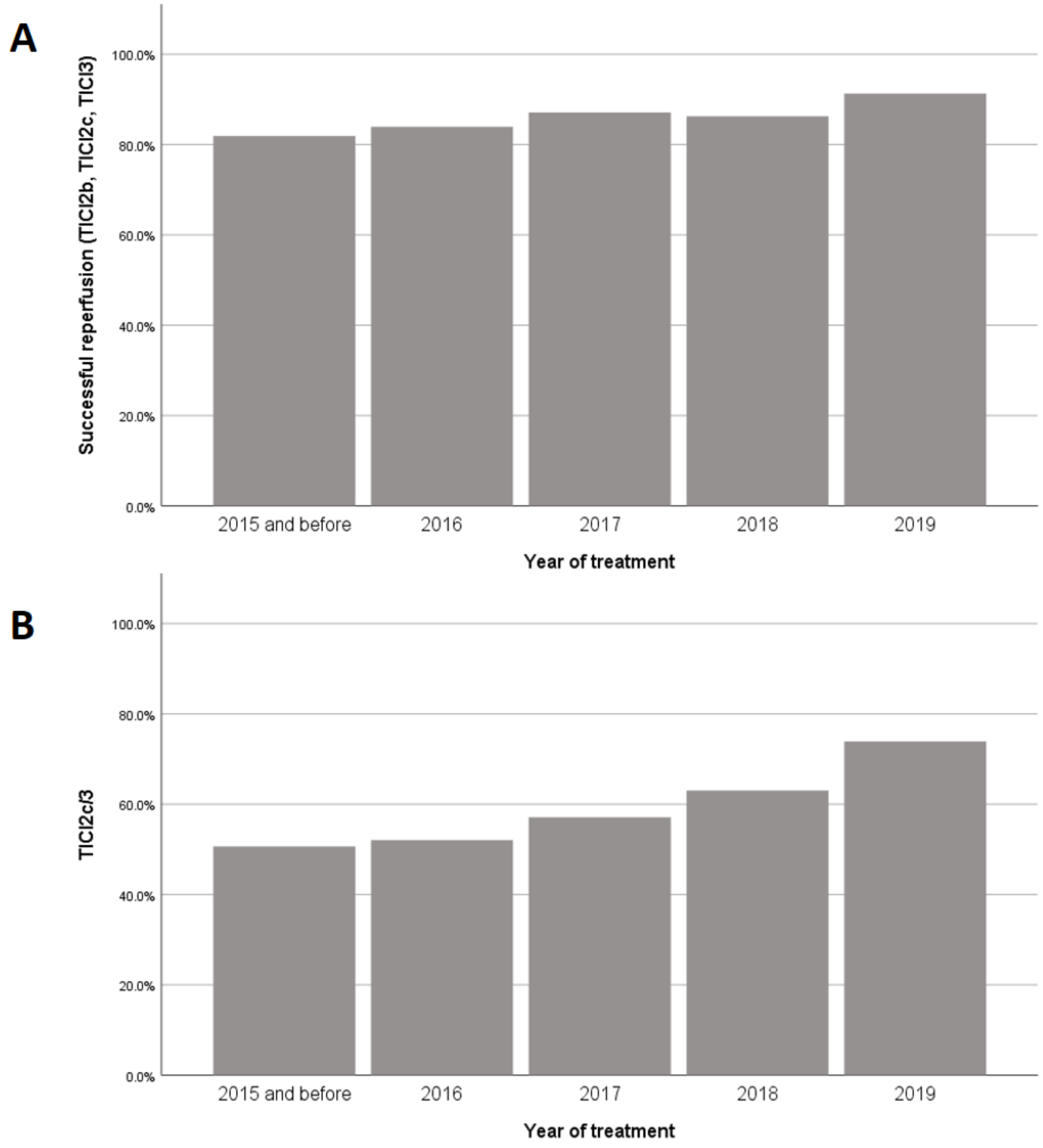

A, there was an increase in successful reperfusion per year after 2015 (OR 1.16, 95%-CI 1.05-1.28 per year). **B**, rates of TICI2c/3 increased per year after 2015 (aOR 1.18, 95%-CI 1.10-1.27 per year). N were 1050, 436, 520, 357 and 23 for 2015 and before, 2016, 2017, 2018 and 2019, respectively. TICI, Thrombolysis in Cerebral Infarction.

Supplementary Figure III – Reperfusion success stratified by interventional technique

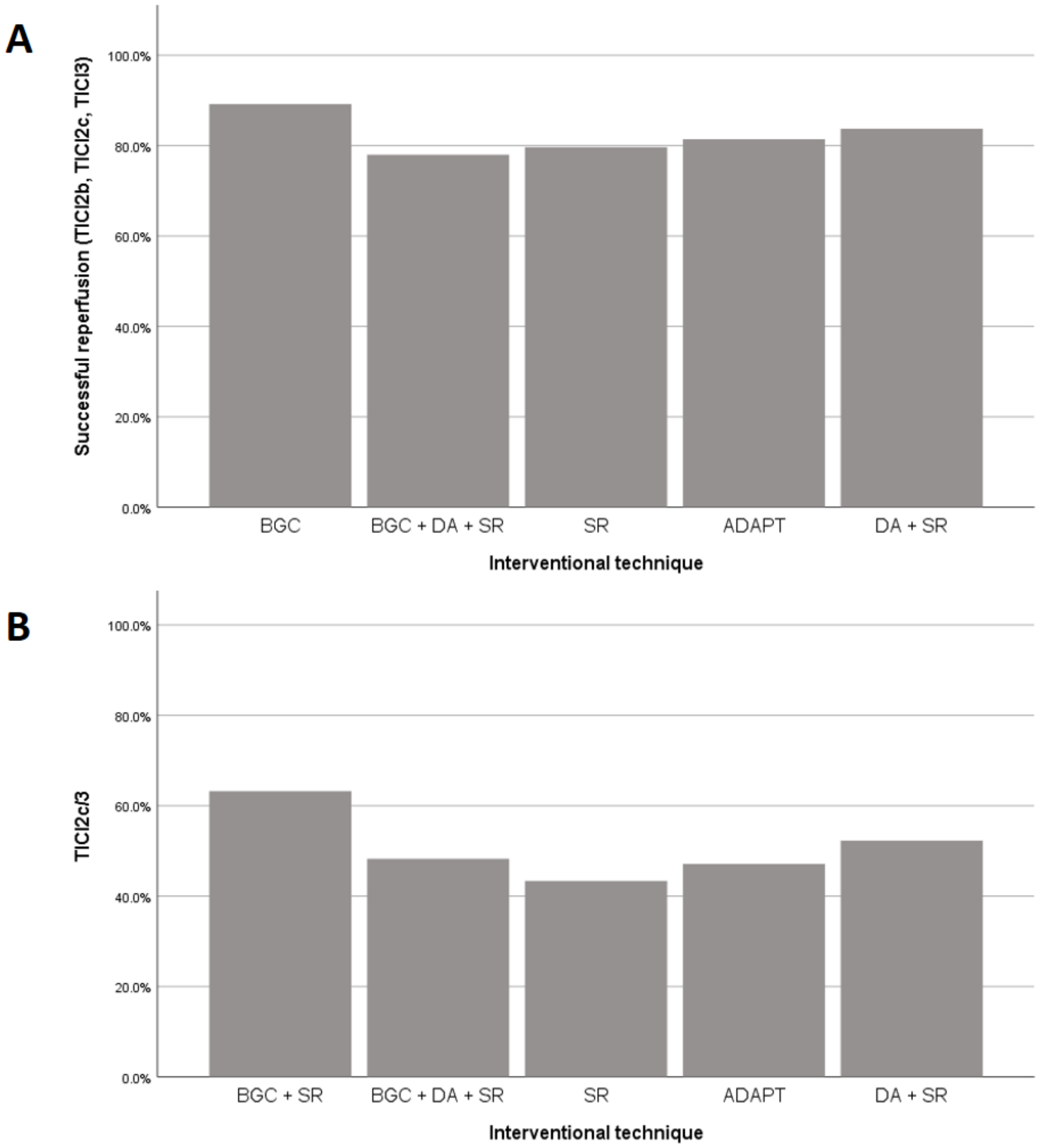

A, Rates of successful reperfusion differed according to the interventional technique applied (chi square P<0.001) with highest rates observed in patients treated with BGC + SR (89.2%); **B**, Rates of TICI2c/3 differed according to the interventional technique applied (chi square P<0.001) with highest rates observed in patients treated with BGC + SR (63.2%). N were 612, 377, 113, 70 and 941 for BGC + SR, BGC + DA + SR, SR, ADAPT and DA + SR, respectively. TICI, Thrombolysis in Cerebral Infarction; BGC, balloon guide catheter; SR, stent-retriever; DA, distal aspiration large bore catheter; ADAPT, A Direct Aspiration First Pass Technique (Aspiration only using large bore catheter, only performed in one center).
